# Be a Mom, a Web-Based Intervention to Promote Positive Mental Health Among Postpartum Women With Low Risk for Postpartum Depression: Exploring Psychological Mechanisms of Change

**DOI:** 10.3389/fpsyt.2021.701107

**Published:** 2021-07-14

**Authors:** Fabiana Monteiro, Marco Pereira, Maria Cristina Canavarro, Ana Fonseca

**Affiliations:** University of Coimbra, Center for Research in Neuropsychology and Cognitive Behavioral Intervention, Faculty of Psychology and Educational Sciences, Coimbra, Portugal

**Keywords:** web-based intervention, randomized controlled trial, positive mental health, self-compassion, postpartum period, multilevel mediation

## Abstract

**Background:** This study explored whether Be a Mom, a brief and unguided cognitive behavioral web-based intervention, was effective in promoting psychological processes (self-compassion, psychological flexibility, emotion regulation) among low-risk postpartum women. Effects of Be a Mom in psychological processes compared with a control group were examined at post-intervention and at 4-months follow-up. Additionally, this work explored whether changes in psychological processes mediated improvements in positive mental health at postintervention.

**Methods:** In total, 367 postpartum women presenting low risk for postpartum depression were randomly assigned to the intervention group (*n* = 191) or to a waiting-list control group (*n* = 176).

**Results:** Compared with the control group, the intervention group reported significantly greater baseline to postintervention increases in self-compassion. No significant effects were found at the 4-month follow-up. Multilevel mediation showed that self-compassion improvements significantly mediated improvements in positive mental health among the intervention group. No significant results were found for psychological flexibility or emotion regulation.

**Conclusions:** This study suggests that Be a Mom has the potential to cultivate self-compassion among low-risk postpartum women and that this may be a key mechanism for promoting positive mental health in this context.

**Clinical Trial Registration:** www.clinicaltrials.gov, identifier: NCT04055974.

## Introduction

The importance of research focused on postpartum women's mental health is highlighted by the long-term impact it has on infant health and development [e.g., (1)], as well as the burden it imposes on economy and society (2, 3), making maternal mental health a health priority. The transition to motherhood constitutes a major life transition as it requires a number of inter- and intrapersonal changes and the need to adapt to numerous demanding tasks and responsibilities, from infant care to financial strains (4, 5). Although there is no consensus on how long the postpartum period is, psychological studies have considered that time frames of up to 1 year after the birth of a child are relevant to study the mother's psychological adjustment to their new role [e.g., (6, 7)]. Given the wide range of stressors that women may experience during this period, this life transition may adversely affect their mental health, which is common to all women in the postpartum period, including those presenting no increased psychosocial risk (8).

Numerous efforts have been made regarding the treatment and prevention of mental disorders during the postpartum period (9); however, from a public health perspective, more needs to be done. Recent studies have highlighted that the promotion of positive mental health is also essential during this period (10, 11) and may have a positive impact on infant development (12). Positive mental health has been considered a multidimensional construct that involves the presence of emotional (positive feelings), psychological (optimal functioning in life) and social (optimal social functioning) well-being (13). Optimal levels of well-being on these three dimensions can be defined as flourishing mental health (13). Positive mental health is an important aspect of coping with adversity as previous results have shown its protective role in the development of mental illness, particularly when exposed to stressful life events [e.g., (14, 15)]. Additionally, high levels of positive mental health have been associated with better psychosocial functioning, better physical health and greater resilience to vulnerabilities and challenges in life (13, 16, 17). More specifically, positive emotions also appear to broaden one's thought–action repertoires and facilitate behavioral flexibility and build personal resources, such as social relationships and resilience (18–20). Considering these benefits and the call for prioritization of research focused on positive mental health (21), increasing studies have focused on directly addressing positive mental health as an intervention outcome [e.g., (22, 23)], including in the perinatal period (10, 24, 25).

Because positive mental health has rarely been studied, there is scarce knowledge on its determinants. Most existing research has focused on sociodemographic and situational variables [e.g., (26, 27)]. However, to build positive mental health, psychological processes and coping skills can be addressed and enhanced through psychological interventions that promote cognitive and emotional resources. This is the case for self-regulatory processes, such as self-compassion, emotion regulation and psychological flexibility, which have often been associated with positive mental health [e.g., (28–31)].

Self-compassion can be defined as an attitude of kindness and acceptance toward oneself when confronted with difficult experiences as opposed to being self-judgmental, feeling isolated and overidentifying with personal difficulties (32). Increasing evidence has suggested that self-compassion could be a meaningful variable in the development and maintenance of positive mental health [e.g., (30, 33, 34)]. Because it invokes kindness, self-compassion can help guard against the self-criticism and guilt that may arise when dealing with the challenges of parenting (35). Indeed, in a sample of postpartum women, recent findings have shown that higher levels of self-compassion increased the likelihood of having higher levels of positive mental health (11). Moreover, a study using a web-based intervention grounded on the compassionate approach found that changes in self-compassion significantly mediated the effect of the intervention on changes in positive mental health among postpartum women (10).

Psychological flexibility refers to the ability to be aware of the present moment and to willingly accept and experience thoughts and feelings that unfold without trying to control or avoid them while acting in a way that is consistent with one's values (36). Previous studies with acceptance-based interventions in general adult population samples have shown that the enhancement of psychological flexibility mediated the effects of the intervention on positive mental health (37, 38). Although still scarce, it has been shown that greater psychological flexibility was a significant contributor to greater positive mental health in postpartum samples (11).

Emotion regulation is a multidimensional construct that can be broadly defined as an individual's ability to identify, to understand, to accept emotional experiences and flexibly modulate emotional responses as situationally appropriate (39). Significant negative associations with positive mental health for maladaptive emotion regulation strategies (e.g., avoidance, rumination) and significant positive associations for adaptive emotion regulation strategies (e.g., cognitive reappraisal, acceptance) have been found (28, 29). Although scarcely studied in relation to positive mental health during the postpartum period, maladaptive emotion regulation strategies have been highlighted as relevant predictors of postpartum depressive symptoms (40, 41). Considering the demands faced by women during the postpartum period, adaptively regulating emotions could be a fundamental aspect of psychological functioning and adjustment.

Considering these findings, it is meaningful to give emphasis to the development of interventions that aim to increase the positive mental health of postpartum women through the fostering of such psychological skills and resources. However, women in the postpartum period face several practical and attitudinal barriers that keep them from seeking traditional face-to-face professional help [e.g., demands associated with infant care, feelings of shame and mental health stigma; (42, 43)]. In this context, eHealth interventions can be particularly helpful, given their accessibility and flexibility and have been increasingly recognized as an effective approach for the delivery of psychological interventions (44, 45). From a public health perspective, these interventions can serve as helpful mental health promotion tools that can be delivered to a broad population and have an important impact on population mental health (46).

Although still limited and very recent, there is preliminary evidence of web-based cognitive behavioral therapy (CBT) interventions as well as compassionate and acceptance-based approaches in enhancing positive mental health in perinatal samples (10, 24). This is the case of Be a Mom, a CBT unguided web-based intervention, which was recently found to be effective in improving positive mental health among low-risk postpartum women (25). Be a Mom incorporates aspects of third-wave CBT approaches and addresses concerns specific to the postpartum period [for the formative evaluation process that informed the design and intervention components see (47)]. Although Be a Mom addresses psychosocial risk factors commonly reported in the perinatal literature (e.g., social support, partner relationship), it also gives particular attention to the development of acceptance- and compassionate-focused skills that promote an adaptive transition to this demanding period. Specifically, Be a Mom touches on the importance of being aware and of non-judgmentally accept the diversity of thoughts and emotions that are common during this period, as well as on the importance of identifying values and goals and engaging in meaningful behaviors according to those values, as opposed to engaging in maladaptive strategies (e.g., experiential avoidance, self-criticism). A previous study found that Be a Mom promoted self-compassion and emotion regulation skills, and this resulted in a significant reduction of depressive symptoms among postpartum women presenting high risk for postpartum depression [PPD] (48). However, we do not know if the same mechanisms of change are responsible for the enhancement of positive mental health among low-risk women.

Therefore, building on these findings [our data derive from a recently performed two-armed pilot randomized controlled trial on the efficacy of Be a Mom in enhancing positive mental health (25)], the objectives of the present study were (1) to examine whether Be a Mom was effective in enhancing psychological processes (self-compassion, psychological flexibility, emotion regulation) among low-risk postpartum women at post-intervention and if these effects were maintained at 4 months follow-up; and (2) to explore whether changes in each psychological process mediated improvements in positive mental health-up.

## Methods

### Setting, Participant Recruitment, and Randomization

This study builds further upon the findings of a recently conducted two-arm, open-label pilot randomized controlled trial (RCT) that showed preliminary evidence of the efficacy of Be a Mom in improving positive mental health among a sample of women presenting low-risk for PPD (25). The study was approved by the Ethics Committee of the Faculty of Psychology and Educational Sciences, University of Coimbra, and it was registered on ClinicalTrials.gov (NCT04055974). Recruitment took place during January 2019 to January 2020 and all participants were recruited online. The access to the intervention was free of cost, and no compensation was given to participants. Outcome variables were assessed at baseline (Time 1—T1), 8 weeks after randomization (Time 2—T2) and 4 months after the postintervention assessment (Time 3—T3) by self-report using the online survey platform Limesurvey. Thorough details of the study design and procedures can be found elsewhere (25).

Briefly, the study was advertised on social media websites through both unpaid cross-posting and paid advertisements. Paid advertisements targeted women aged between 18 and 45 years old with interests in maternity and newborn topics. Participants who clicked on the advertised link were directed to a page containing information about the aims and procedures of the Be a Mom trial, the participants' and researchers' roles and the voluntary and anonymous nature of participation. After giving online informed consent (by clicking on the option “I understand and accept the conditions of the study”), participants answered a set of questions to assess eligibility criteria. The eligibility criteria of the study were as follows: to be in the early postpartum period (up to 3 months postpartum); age ≥18 years; to present low risk for PPD (Postpartum Depression Predictors Inventory-Revised <5.5); to have internet access at home; to be a resident of Portugal; and to understand Portuguese. Participants were excluded if they had a serious medical condition (physical or psychiatric) or if the infant had a serious health condition (both self-reported).

Randomization was conducted using a computerized random number generator (allocation ratio 1:1). The first author was responsible for the enrollment and assignment of participants and the last author was responsible for randomization. Participants were not blinded to the assigned group.

*A priori* calculations suggested that a sample size of at least 200 participants at postintervention assessment was needed to assess the efficacy of Be a Mom in promoting psychological resources [detecting a small effect size (*d* = 0.10) with a statistical power of 0.80 in a two-tailed test, *p* < 0.05]. Considering the dropout rate of approximately 35% in the pilot study of Be a Mom (49), at least 350 participants were needed for randomization.

### Intervention

Participants in the intervention arm were invited to a password-protected website with the intervention (beamom.pt). The intervention entails five modules, each averaging 45 min in duration. The duration of the intervention was 5 weeks, but participants were given 8 weeks after randomization to complete the five modules. Those who registered on the website were contacted via telephone to confirm proper use of the platform and to facilitate intervention adherence. Participants were sent an email with the postintervention assessment 8 weeks after randomization.

Participants in the WLC arm were informed that they would receive access to Be a Mom at the end of the study and were also asked to complete the postintervention assessment protocol 8 weeks after randomization. The T3 protocol assessment was sent to participants of both groups 4 months after postintervention. Participants in both groups had unrestricted access to usual treatment options. The proportion of women who had psychological/psychiatric treatment after the baseline assessment was similar in both groups at T2 (intervention group: *n* = 8, 7.7% vs. WLC group: *n* = 7, 4.9%, χ^2^ = 0.85, *p* = 0.356) and T3 (intervention group: *n* = 6, 7.1% vs. WLC group: *n* = 9, 6.7%, χ^2^ = 0.01, *p* = 0.922).

### Measures

Participants answered a self-reported questionnaire developed by the researchers, including questions about sociodemographic (e.g., age, marital status, educational level) clinical (e.g., psychopathological history) and infant-related data (e.g., infant age, gestational weeks at birth).

### Risk for PPD

The Portuguese Version (PV) of the Postpartum Depression Predictors Inventory-Revised [PDPI-R; (50)] was used to identify women presenting low risk for PPD. Examples of the factors assessed include marital and socioeconomic status, prenatal depression and anxiety, history of depression, social support, marital satisfaction, life stress and infant temperament. The PDPI-R comprises 39 items answered on a dichotomous scale (yes vs. no, except for the first two items in which participants report their marital and socioeconomic status). The PDPI-R total score ranges from 0 to 39. Higher scores indicate increased risk for PPD. In Portuguese validation studies, a score of 5 or lower is indicative of lower PPD risk (51).

### Positive Mental Health

Positive mental health was assessed using the PV of the Mental Health Continuum-Short Form [MHC-SF; (52)]. The MHC-SF comprises 14 items divided into three dimensions: emotional (three items; e.g., “During the past month, how often did you feel happy?”), social (five items; e.g., “During the past month, how often did you feel that you belonged to a community?”) and psychological well-being (six items; e.g., “During the past month, how often did you feel that you liked most parts of your personality?”). Each item is rated on a six-point response scale from 0 (*never*) to 5 (*every day*). Scores on the MHC-SF range from 0 to 70, and higher scores indicate better positive mental health. The MHC-SF also allows a classification of three positive mental health categories: flourishing mental health, moderate mental health and languishing mental health. A classification of flourishing entails scoring 4 or 5 on at least one item of the emotional well-being subscale in combination with a 4 or 5 score on at least six items of the social and psychological well-being subscales. Languishing is classified as a score of 0 or 1 on at least one item of the emotional well-being subscale and at least six items of the social and psychological well-being subscales. Those who did not fit these criteria were classified as moderately mentally healthy. In this study, we categorized participants as flourishing and not flourishing (including both languishers and those with moderate mental health). In Portuguese psychometric studies, only the use of the total score was recommended, as no adequate support was found for the use of the subscales as measures of distinct dimensions (52). Cronbach's alpha values of this study ranged from 0.90 (intervention group—T1) to 0.93 (intervention group—T2).

### Process Variables

The PV of the Self-Compassion Scale—Short Form [SCS-SF; (53)] was used to assess women's self-compassion levels. The SCS-SF is a self-report measure comprising 12 items (e.g., “I'm disapproving and judgmental about my own flaws and inadequacies”) answered on a five-point response scale ranging from 1 (*almost never*) to 5 (*almost always*). Higher scores indicate higher levels of self-compassion. In our sample, Cronbach's alpha values ranged from 0.85 (intervention group—T3) to 0.92 (control group—T2).

The PV of the Acceptance and Action Questionnaire-II [AAQ-II; (54)] was used to assess psychological flexibility. Participants were asked to rate each of the seven items (e.g., “Worries get in the way of my success”) on a seven-point response scale ranging from 1 (*never true*) to 7 (*always true*). Higher scores are reflective of greater psychological flexibility. In our sample, Cronbach's alpha values ranged from 0.87 (control group—T2) to 0.93 (intervention group—T2).

The PV of the Difficulties in Emotion Regulation Scale-Short Form [DERS-SR; (55)] was used to assess emotion regulation skills. The DERS-SR comprises 18 items (e.g., “I pay attention to how I feel”) answered on a five-point scale ranging from 1 (*almost never*) to 5 (*almost always*). Higher scores suggest more adaptive emotion regulation skills. In this study, Cronbach's alpha values ranged from 0.88 (intervention group—T1) to 0.92 (control group—T3).

### Data Analysis

Statistical analyses were conducted on all randomized participants, according to the intention-to-treat principles following the CONSORT statement (56). Data were analyzed using the *Statistical Package for Social Sciences* (IBM SPSS, version 23.0). Descriptive statistics were computed for sample characterization and comparison tests (*t*-test and chi-squared) were performed to compare the intervention and the WLC group as well as completers and dropouts (i.e., those who did not complete T2 and T3 assessments) in terms of background characteristics. Missing endpoints at T2 ranged from 119/367 (32.4%) on MHC-SF to 127/367 (34.6%) on SCS-SF and from 162/367 (44.1%) on AAQ-II to 170/367 (46.3%) on SCS-SF at T3.

Linear mixed models with an autoregressive covariance matrix were used to determine the effects of the intervention from baseline to postintervention on psychological processes. To examine if these effects were maintained after 4 months, linear mixed models were also performed. When conducting mixed models, all available data are used to obtain parameter estimates with small bias in the presence of data missing completely at random or missing at random (T2 Little's MCAR test χ^2^ = 30.24, *p* = 0.995; T3 Little's MCAR test χ^2^ = 23.03, *p* = 0.576) (57). Group, time and time by group interaction and covariates (variables presenting significant differences between intervention and control groups at baseline and between completers and dropouts at T2 and T3: previous history of psychopathology, infant's age, education and category of positive mental health) were fitted as fixed effects. Participant was included as a random intercept.

**Figure 1:**
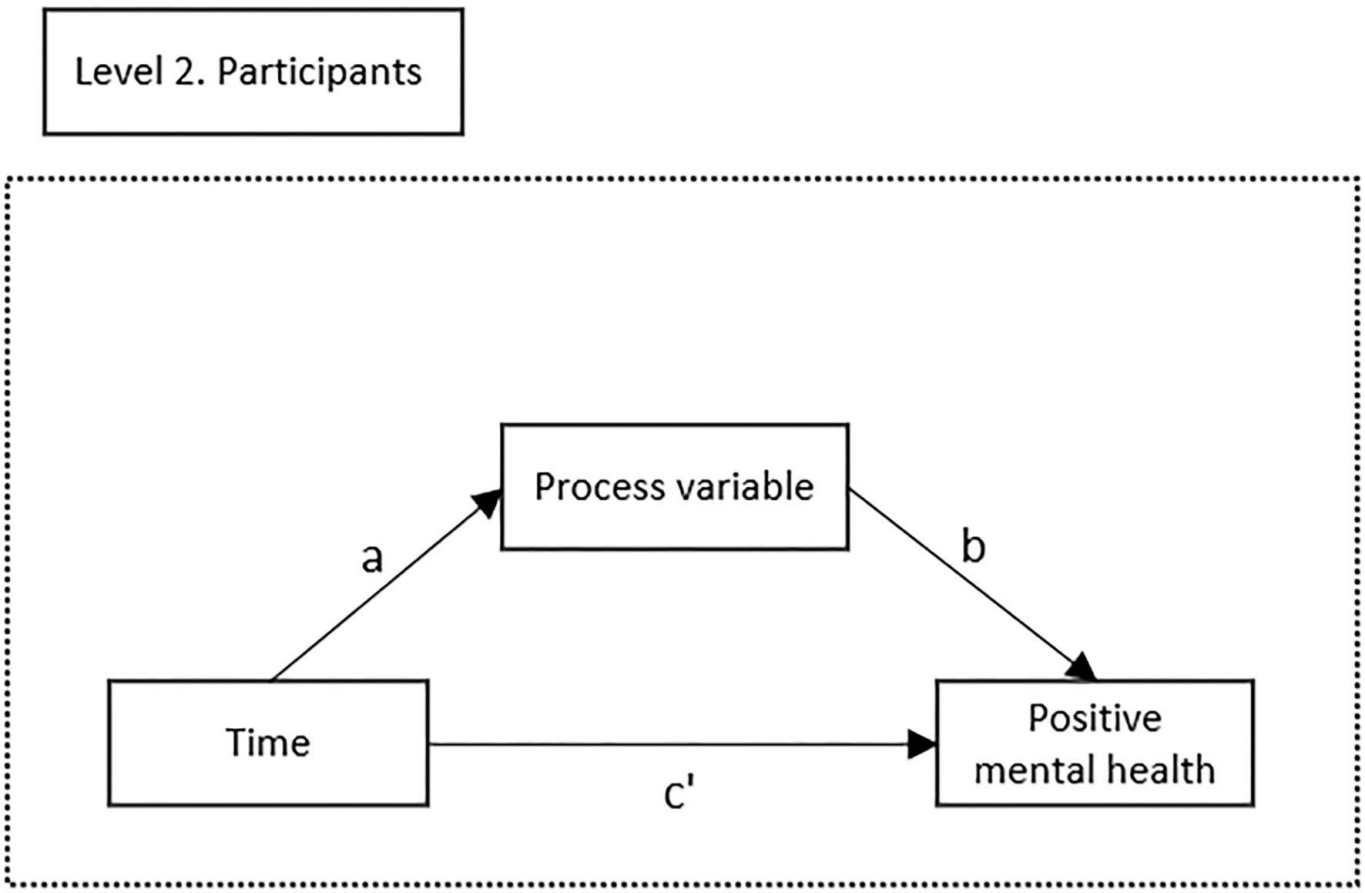
Illustration of the multilevel mediation model. Path a represents the effect of time on the process variable. Path b represents the change in the process variable when positive mental health changes with one unit when time is held constant. Path c′ represents the direct effect of time on positive mental health when controlling for the effect of changes in the process variable. Path a^*^b represents the within-group indirect effect of time on positive mental health represented by changes in the process variable.

To examine if changes in process variables (from T1 to T2) statistically mediated the effect of Be a Mom on T1 to T2 change in positive mental health, 1-1-1 multilevel mediation models were estimated using MLmed (58), a SPSS macro that tests for mediation in multilevel data based on procedures suggested by Bauer, Preacher (59). In line with the intention-to-treat principles, multilevel mediation allows all participants to be included, irrespective of missing data. It extends on classic mediation models to account for the nested nature of repeated measures data (i.e., time nested within individuals). Thus, the relationships between variables are calculated within each individual over time and then aggregated across the sample to represent the overall relationship between those variables. Using this approach, all three proposed mediators (self-compassion, psychological flexibility and emotion regulation) were tested individually for each study group. Parameters were estimated using restricted maximum likelihood estimation. Only within-group effects were analyzed. Figure 1 illustrates the 1-1-1 multilevel mediation model.

## Results

### Participant Characteristics

**Figure 2:**
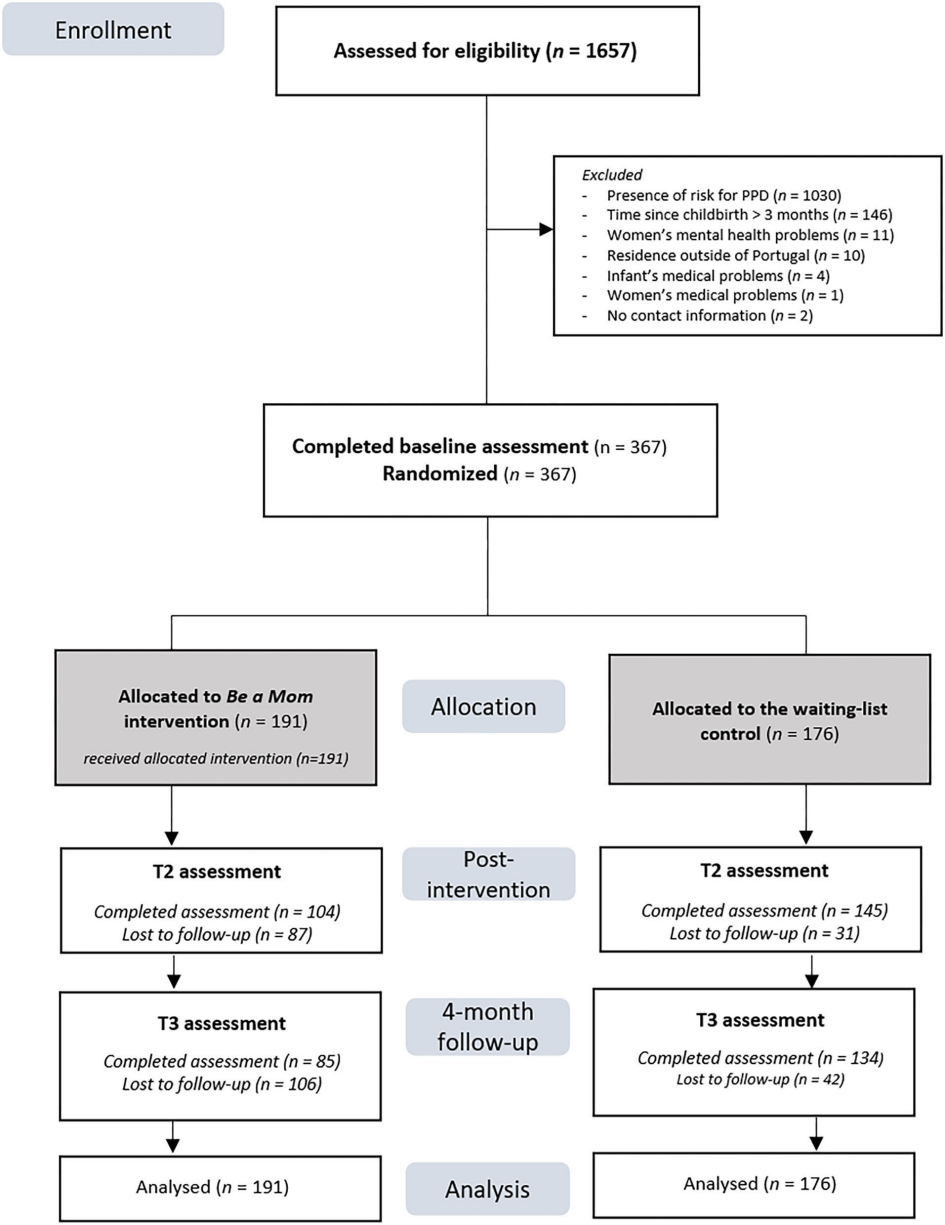
Flowchart of the participants in the study.

In total, 1,657 women were screened for eligibility. A majority of those interested presented risk factors for PPD and were allocated to a different RCT aiming to assess the effectiveness of Be a Mom in preventing PPD. Of the 498 women who were sent the baseline assessment, 403 completed the set of questionnaires and were randomized to either the Be a Mom group (*n* = 212) or the WLC group (*n* = 191). See Figure 2 for participant flow.

Table 1 presents the baseline descriptive characteristics of the intervention and control groups. Significant differences were found for history of psychopathology and presence of flourishing between both groups. A significantly higher proportion of participants in the intervention group had a previous history of psychopathology (25.1 vs. 14.2%, χ^2^ = 6.86, *p* = 0.009) and were not flourishing (42.9 vs. 33%, χ^2^ = 3.86, *p* = 0.049) compared with the WLC group.

**Table 1:** Participants' background characteristics at baseline.

	**Intervention Group (*n* = 191) *M* (*SD*)/*n* (*%*)**	**Control Group (*n* = 176) *M* (*SD*)/*n* (*%*)**	***t/**χ^2^*****
Age	32.97 (4.04)	33.03 (4.43)	−0.14
Marital status			0.53
Married/co-habiting	183 (95.8)	170 (96.6)	
Single	4 (2.1)	2 (1.1)	
In a relationship (without living together)	4 (2.1)	4 (2.3)	
Employment status			3.35
Employed	176 (92.1)	170 (96.6)	
Not currently working	15 (7.9)	6 (3.4)	
Educational level			5.66
Up to the 9th grade	2 (1.0)	4 (2.3)	
High school	30 (15.7)	26 (14.8)	
Bachelor's degree	83 (43.5)	58 (33)	
Master's or Doctorate	76 (39.8)	88 (50)	
Household monthly income			4.92
Less than 580€	8 (4.2)	9 (5.1)	
580€-1000€	88 (46.1)	80 (45.5)	
1000€-2000€	87 (45.5)	70 (39.8)	
More than 2000€	8 (4.2)	17 (9.7)	
Residence			1.06
Urban	141 (73.8)	138 (78.4)	
Rural	50 (26.2)	38 (21.6)	
Psychopathology history			6.86^*^
Yes	48 (25.1)	25 (14.2)	
No	143 (74.9)	151 (85.8)	
Positive mental health			3.86^*^
Flourishing	109 (57.1)	118 (67)	
Not flourishing	82 (42.9)	58 (33)	
Infant's age (in months)	1.89 (0.94)	1.87 (1.32)	0.16
Infant's gestational weeks	38.89 (1.64)	38.95 (1.77)	−0.33
Primiparous	140 (73.3)	122 (69.3)	0.71

* *p <0.05*.

Of the 367 participants, 249 (67.8%) completed the postintervention assessment and 219 (59.7%) completed the 4-month follow-up. The intervention arm presented a significantly higher loss to postintervention than the WLC arm (intervention group: *n* = 87, 45.5% vs. control group: *n* = 31, 17.6%, χ^2^ = 32.77, *p* < 0.001). Infants of the participants who dropped out from the study were significantly older than the infants of those who completed the postintervention assessment (*M* = 2.12 months, *SD* = 0.96 vs. *M* = 1.77 months, *SD* = 1.20, *t*(365) = −2.77, *p* = 0.006). At T3, the intervention group also presented a significantly higher loss to follow-up compared to the WLC group (intervention group: *n* = 134, 61.2% vs. control group: *n* = 42, 28.4%, χ^2^ = 38.09, *p* < 0.001). Participants who did not complete T3 assessment had significantly older infants (*M* = 2.05 months, *SD* = 0.96 vs. *M* = 1.71 months, *SD* = 0.91, *t*(365) = −3.48, *p* = 0.001) and lower education (proportion of dropouts with a bachelor's degree: *n* = 47, 31.8% vs. completers: *n* = 93, 42.5%, χ^2^ = 9.19, *p* = 0.027) than those who completed the 4-month follow-up assessment.

### Change in Process Variables in the Intervention and Control Groups: Examining the Enhancement of Process Variables at Post-intervention and Its Maintenance at 4 Months Follow-Up

To examine changes in process variables between baseline and post-intervention both in the intervention and control groups, a multilevel model was estimated. Table 2 presents the estimated marginal means of the process variables and fixed effects for time, group and the time x group interaction as well as for covariates (psychopathology history, infant's age and category of positive mental health at baseline).

**Table 2:** Estimated marginal means and fixed effects for psychological processes at postintervention.

	**Group**	**Time 1 *M* (*SE*)**	**Time 2 *M* (*SE*)**	**Effect**	***B* (*SE*)**	**95% CI**	***p***
Self-compassion	Intervention	41.72 (0.55)	44.09 (0.68)	Time	−2.37 (0.58)	(−3.52, −1.23)	<0.001
	Control	41.72 (0.58)	42.07 (0.61)	Group	−2.03 (0.92)	(−3.82, −0.23)	0.027
				Time x Group	2.03 (0.76)	(0.53, 3.54)	0.008
				Psychopathology history	4.45 (0.95)	(2.58, 6.31)	<0.001
				MHC-SF baseline category	6.61 (0.78)	(5.08, 8.13)	<0.001
				Infant's age	0.51 (0.33)	(−0.13, 1.16)	0.120
Psyhological flexibility	Intervention	39.22 (0.50)	39.86 (0.61)	Time	−0.64 (0.52)	(−1.66, 0.37)	0.214
	Control	39.17 (0.52)	39.36 (0.55)	Group	−0.50 (0.82)	(−2.12, 1.12)	0.544
				Time x Group	0.45 (0.68)	(−0.89, 1.79)	0.505
				Psychopathology history	4.80 (0.86)	(3.10, 6.49)	<0.001
				MHC-SF baseline category	5.38 (0.70)	(3.99, 6.77)	<0.001
				Infant's age	−0.30 (0.30)	(−0.88, 0.29)	0.320
Emotion regulation skills	Intervention	73.98 (0.69)	75.39 (0.87)	Time	−0.42 (0.82)	(−2.03, 1.20)	0.614
	Control	73.11 (0.72)	72.94 (0.77)	Group	−2.46 (1.17)	(−4.76, −0.16)	0.036
				Time x Group	0.59 (1.08)	(−1.54, 2.73)	0.584
				Psychopathology history	4.04 (1.16)	(1.76, 6.32)	<0.001
				MHC-SF baseline category	6.50 (0.95)	(4.63, 8.36)	<0.001
				Infant's age	−0.08 (0.40)	(−0.87, 0.70)	0.836

*MHC-SF, mental health continuum-short form*.

Significant effects of time, group and time x group interaction were found for self-compassion, with women in the intervention group reporting a higher increase in self-compassion levels when compared with participants in the WLC group.

No interaction effects of time and group were found for the remaining variables. Regarding emotion regulation skills, a significant effect of group was found, with the intervention group exhibiting overall higher emotion regulation skills. Although no significant time x group interactions were found for psychological flexibility and emotion regulation skills, Table 2 shows a greater increase in these psychological processes from T1 to T2 in the intervention group.

Concerning the maintenance at 4 months follow-up, there were no statistically significant differences in the rate of change for process variables between the intervention and control groups at T3 (see Table 3). The overall higher estimated means in the intervention group suggest that gains in self-compassion for participating in Be a Mom were relatively maintained at 4 months postintervention.

**Table 3:** Estimated means and fixed effects for psychological processes at 4-months follow-up.

	**Group**	**Time 2 *M* (*SE*)**	**Time 3 *M* (*SE*)**	**Effect**	***B* (*SE*)**	**95% CI**	***p***
Self-compassion	Intervention	44.55 (0.77)	44.05 (0.83)	Time	0.50 (0.64)	(−0.77, 1.76)	0.438
	Control	42.19 (0.63)	42.22 (0.65)	Group	−1.83 (1.06)	(−3.91, 0.25)	0.085
				Time x Group	−0.53 (0.81)	(−2.13, 1.07)	0.516
				Psychopathology history	3.60 (1.12)	(1.39, 5.81)	0.002
				MHC-SF baseline category	−6.06 (0.96)	(−7.96, −4.17)	<0.001
				Education	0.76 (0.64)	(−0.49, 2.01)	0.232
				Infant's age	0.48 (0.51)	(−0.51, 1.48)	0.339
Psyhological flexibility	Intervention	40.14 (0.69)	39.87 (0.71)	Time	0.27 (0.65)	(−1.00, 1.55)	0.673
	Control	39.42 (0.58)	39.10 (0.60)	Group	−0.77 (0.97)	(−2.67, 1.13)	0.426
				Time x Group	0.05 (0.82)	(−1.58, 1.68)	0.951
				Psychopathology history	3.59 (1.00)	(1.63, 5.55)	<0.001
				MHC-SF baseline category	−3.47 (0.86)	(−5.16, −1.78)	<0.001
				Education	0.52 (0.57)	(−0.60, 1.64)	0.358
				Infant's age	0.07 (0.45)	(−0.82, 0.95)	0.883
Emotion regulation skills	Intervention	76.21 (1.03)	75.51 (1.09)	Time	0.70 (0.77)	(−0.83, 2.22)	0.368
	Control	73.79 (0.86)	73.68 (0.88)	Group	−1.84 (1.41)	(−4.61, 0.94)	0.195
				Time x Group	−0.59 (0.98)	(−2.53, 1.35)	0.550
				Psychopathology history	4.31 (1.53)	(1.30, 7.32)	0.005
				MHC-SF baseline category	−5.17 (1.32)	(−7.77, −2.57)	<0.001
				Education	1.12 (0.87)	(−0.60, 2.84)	0.200
				Infant's age	−0.60 (0.69)	(−1.96, 0.76)	0.383

*MHC-SF, mental health continuum-short form*.

### The Role of Psychological Processes in the Improvement of Positive Mental Health From Baseline to Post-intervention

Tables 4, 5 present the path coefficients, direct and indirect effects of the multilevel mediation models for self-compassion, psychological flexibility and emotion regulation skills in the intervention and control group, respectively.

**Table 4:** Multilevel coefficients and indirect effects for mediation of psychological processes on positive mental health in the intervention group.

	***a***	***b***	**Direct effect *c'***	**Indirect effect**	
				***ab***	**95% CI**
Self-compassion	2.24^***^	0.35^**^	1.26	0.78^*^	(0.24, 1.48)
Psychological flexibility	0.53	0.31^*^	1.88^**^	0.16	(−0.13, 0.58)
Emotion regulation skills	0.28	0.16	1.98^**^	0.04	(−0.21, 0.35)

* *p <0.05*;

** *p <0.01*;

*** *p <0.001*.

**Table 5:** Multilevel coefficients and indirect effects for mediation of psychological processes on positive mental health in the control group.

	***a***	***b***	**Direct effect *c'***	**Indirect effect**	
				***ab***	**95% CI**
Self-compassion	0.34	0.23*	0.61	0.08	(−0.16, 0.40)
Psychological flexibility	0.18	0.56***	0.56	0.10	(−0.40, 0.64)
Emotion regulation skills	−0.19	0.25**	0.75	−0.05	(−0.44, 0.34)

*p <0.05; **p <0.01; ***p <0.001.

Among the intervention group, there was a significant increase in self-compassion levels over time. Regarding indirect effects, the analyses revealed that baseline to postintervention increases in self-compassion were a significant mediator of change in positive mental health over that period.

Concerning the WLC group, the results showed that there were no significant increases in psychological processes over time. Regarding direct and indirect effects, no significant relationships were found.

## Discussion

While interest in web-based interventions aimed at improving positive mental health is increasing, direct empirical evidence supporting the mechanisms of change under these interventions is lacking, particularly for the perinatal period. Understanding treatment processes and researching and intervening at such level is essential and has been considered a promising path forward for intervention science (60). Therefore, while there is evidence of the efficacy of Be a Mom in improving positive mental health (25), this study aimed to explore the mechanisms underlying this improvement. Overall, our results showed that participating in Be a Mom significantly increased self-compassion levels and that this increase was associated with the improvement in positive mental health.

Be a Mom's efficacy in significantly enhancing self-compassion levels and emotion regulation skills was previously established among a high-risk sample of postpartum women (48). This study demonstrates that this intervention is also effective in promoting self-compassion levels among low-risk postpartum women. Although no significant differences were found between the two groups at the 4-month follow-up, the results suggest that the increased levels in self-compassion among Be a Mom participants were maintained over that period. The postpartum is a time characterized by incongruences between the expectations and realities of motherhood (61) that are strongly influenced by the ideology of perfect motherhood, which can lead women to self-criticize themselves and to a sense of shame and guilt (62). When using Be a Mom, participants were taught about the myth of perfect motherhood and the pervasive role of self-criticism when dealing with postpartum stressors (e.g., infant care, breastfeeding). They were offered exercises aimed at promoting acts of compassion inwards when considering the maternal role and at promoting a sense of common humanity in which women realize that their suffering is a normal part of the human (motherhood) experience and that they are not alone in their suffering and self-judgment (32).

Although emotion regulation skills and psychological flexibility increased at postintervention, contrary to our expectations, the results were not significantly different from those reported in the WLC group. However, our results demonstrated a trend of a greater increase in the intervention group in both variables. In the case of emotion regulation skills, mean estimates showed that from T1 to T2, there was an increase in the intervention group but a decrease in the control group. The higher levels of emotion regulation skills in the intervention group are also reported at T3, suggesting that it is possible that participating in Be a Mom may have contributed to more adaptive emotion regulation strategies. Nevertheless, it is possible that Be a Mom is not as effective in fostering emotion regulation skills among low-risk women. It has been previously suggested that at-risk mothers could obtain more benefits from postpartum interventions as there is generally more room for improvement (63).

Likewise, Be a Mom did not significantly improve psychological flexibility, which is in line with what was previously found among a high-risk sample (48). It is possible that this failure in finding a significant improvement could in part be due to the instrument that was used to measure psychological flexibility. Although the AAQ-II is the most used self-report measure of psychology flexibility, it has been shown that, compared to other measures, the AAQ-II is less sensitive to treatment change (64). Indeed, previous intervention studies using an Acceptance and Commitment Therapy approach have not found significant results when considering AAQ-II scores (22, 65). Using a different measure of psychological flexibility or measuring associated constructs such as coping flexibility, [i.e., the capacity to discontinue an ineffective coping strategy and carry out an alternative one, (66)] could help determine whether Be a Mom significantly promotes this psychological process.

The results of the multilevel mediation analyses between baseline and postintervention provide valuable information on the relation between the psychological processes targeted in Be a Mom and positive mental health. Specifically, self-compassion fully statistically mediated the improvement of positive mental health among the intervention group. This is consistent with the results of a previous study using a web-based intervention for postpartum women (10) and with studies that have suggested that self-compassion is a particularly essential variable for a healthy psychological adjustment to the postpartum period (11, 67). Self-compassion appears to support positive mental health, protecting against the oftentimes stressful and negative impact of the transition to motherhood. This significant association was only demonstrated in the intervention group, suggesting that participating in Be a Mom promoted self-compassion, which, in turn, contributed to the improvement of positive mental health. Thus, promoting self-compassion during the postpartum period seems to be very valuable to a better positive mental health and this can be achieved using brief and unguided web-based interventions, grounded on CBT and third-wave CBT approaches.

Psychological flexibility and emotion regulation skills did not significantly contribute to the improvement of positive mental health. Although there is evidence of psychological flexibility as a significant mediator of positive mental health improvement among acceptance and commitment therapy-based interventions [e.g., (37)], other studies have found that increases in psychological flexibility were not related to positive mental health [e.g., (68, 69)]. Nevertheless, when comparing multilevel mediation results of the intervention and control groups, the results suggest that significant direct effects for psychological flexibility and emotion regulation skills were only found for the intervention group. Therefore, it seems that participating in Be a Mom was beneficial for the psychological health of these women.

Although this study provides relevant contributions to the area of interventions for maternal mental health, we acknowledge some limitations. First, because of the self-selected convenience sample, caution is needed in generalizing these findings. Second, there was a high attrition rate over time. This is in line with previous studies using unguided web-based interventions, particularly in the perinatal period (70), which is recognized as a demanding time. Following the intention-to-treat principles, we used linear mixed methods and multilevel mediation to handle missing data and minimize the effect of study dropouts. However, this could have influenced the results. Regarding mediation analyses, we must acknowledge that we cannot assume that the investigated variables are independent from each other or that they only form unidirectional and linear relationships. Although our findings indicated that self-compassion plays a central role in the enhancement of positive mental health among postpartum women who use Be a Mom, they do not establish definitive evidence of causality (71). Moreover, we cannot exclude the possibility that other unknown variables could have affected positive mental health.

Extensive research on the perinatal period has focused on at-risk women and the prevention of psychopathology, particularly PPD (9). However, it has been proposed that only targeting at-risk groups is not sufficient (46) and that, from a population health perspective, targeting the promotion of positive mental health among low-risk women is also essential. This study emphasizes that in order to achieve this goal, intervention research cannot mainly focus on diminishing psychosocial risk but instead must concentrate on investigating the influence of core psychological processes that can contribute to a better adjustment to this period. Particularly, this study is one of the first to assess psychological mediators of change during the course of a short and unguided web-based intervention for the promotion of postpartum women's mental health. Our study suggests that fostering a more compassionate, non-judgmental and accepting attitude toward the diversity of difficult internal and external experiences during the postpartum period—a significant period with implications for the overall health of women and infants—is especially important for improving positive mental health among low-risk postpartum women. In turn, promoting positive mental health and going beyond a focus on simply eliminating mental illness could result in more satisfying and fulfilling lives and set women on trajectories of growth that could consequential lead to the building of a wide range of personal resources (19, 20).

## Data Availability Statement

The data analyzed in this study is subject to the following licenses/restrictions: The datasets that support the findings of this study are available upon reasonable request from the corresponding author (Fabiana Monteiro). The data are not publicly available due to them containing information that could compromise the privacy of research participants. Requests to access these datasets should be directed to Fabiana Monteiro, fgmonteiro.91@gmail.com.

## Ethics Statement

The studies involving human participants were reviewed and approved by the Ethics Committee of the Faculty of Psychology and Educational Sciences, University of Coimbra. The patients/participants provided their written informed consent to participate in this study.

## Author Contributions

Conceptualization: FM, MC, and AF. Methodology: FM, MP, and AF. Formal analysis and investigation: FM and AF. Supervision and Writing—review and editing: MP, MC, and AF. Writing—original draft: FM. All authors have read and agreed to the published version of the manuscript.

## Conflict of Interest

The authors declare that the research was conducted in the absence of any commercial or financial relationships that could be construed as a potential conflict of interest.

## References

[1] Slomian J Honvo G Emonts P Reginster JY Bruyere O. Consequences of maternal postpartum depression: a systematic review of maternal and infant outcomes. Womens Health. (2019) 15:1745506519844044. 10.1177/1745506519844044 31035856 PMC6492376

[2] Bauer A Knapp M Parsonage M. Lifetime costs of perinatal anxiety and depression. J Affect Disord. (2016) 192:83–90. 10.1016/j.jad.2015.12.005 26707352

[3] Ladd C Rodriguez McCullough N Carmaciu C. Perinatal mental illness. InnovAiT. (2017) 10:653–8. 10.1177/1755738017722171

[4] Kanotra S D'Angelo D Phares TM Morrow B Barfield WD Lansky A. Challenges faced by new mothers in the early postpartum period: an analysis of comment data from the 2000 Pregnancy Risk Assessment Monitoring System (PRAMS) survey. Matern Child Health. (2007) 11:549–58. 10.1007/s10995-007-0206-3 17562155

[5] Jevitt CM Groer MW Crist NF Gonzalez L Wagner VD. Postpartum stressors: a content analysis. Issues in Mental Health Nursing. (2012) 33:309–18. 10.3109/01612840.2011.653658 22545638

[6] O'Hara MW McCabe JE. Postpartum depression: current status and future directions. Annu Rev Clin Psychol. (2013) 9:379–407. 10.1146/annurev-clinpsy-050212-185612 23394227

[7] Gavin NI Gaynes BN Lohr KN Meltzer-Brody S Gartlehner G Swinson T. Perinatal depression: a systematic review of prevalence and incidence. Obstet Gynecol. (2005) 106(5 Pt 1):1071–83. 10.1097/01.AOG.0000183597.31630.db 16260528

[8] Murphey C Carter P Price LR Champion JD Nichols F. Psychological distress in healthy low-risk first-time mothers during the postpartum period: an exploratory study. Nurs Res Pract. (2017) 2017:8415083. 10.1155/2017/8415083 28191350 PMC5278222

[9] Sockol LE. A systematic review of the efficacy of cognitive behavioral therapy for treating and preventing perinatal depression. J Affect Disord. (2015) 177:7–21. 10.1016/j.jad.2015.01.052 25743368

[10] Gammer I Hartley-Jones C Jones FW. A randomized controlled trial of an online, compassion-based intervention for maternal psychological well-being in the first year postpartum. Mindfulness. (2020) 11:928–39. 10.1007/s12671-020-01306-9

[11] Monteiro F Fonseca A Pereira M Canavarro MC. Is positive mental health and the absence of mental illness the same? Factors associated with flourishing and the absence of depressive symptoms in postpartum women. J Clin Psychol. (2020) 77:629. 10.1002/jclp.23081 33098665

[12] Phua DY Kee M Koh DXP Rifkin-Graboi A Daniels M Chen H. Positive maternal mental health during pregnancy associated with specific forms of adaptive development in early childhood: evidence from a longitudinal study. Dev Psychopathol. (2017) 29:1573–87. 10.1017/S0954579417001249 29162171

[13] Keyes CL. Mental illness and/or mental health? Investigating axioms of the complete state model of health. J Consult Clin Psychol. (2005) 73:539–48. 10.1037/0022-006X.73.3.539 15982151

[14] Truskauskaite-Kuneviciene I Kazlauskas E Ostreikaite-Jurevice R Brailovskaia J Margraf J. Positive mental health and adjustment following life-stressors among young adults. Curr Psychol. (2020). 10.1007/s12144-020-00714-3

[15] Brailovskaia J Teismann T Margraf J. Positive mental health, stressful life events, and suicide ideation. Crisis. (2020) 41:383–8. 10.1027/0227-5910/a000652 32036702

[16] Keyes CL Simoes EJ. To flourish or not: positive mental health and all-cause mortality. Am J Public Health. (2012) 102:2164–72. 10.2105/AJPH.2012.300918 22994191 PMC3477942

[17] Dyrbye LN Harper W Moutier C Durning SJ Power DV Massie FS. A multi-institutional study exploring the impact of positive mental health on medical students' professionalism in an era of high burnout. Acad Med. (2012) 87:1024–31. 10.1097/ACM.0b013e31825cfa35 22722352

[18] Cohn MA Fredrickson BL Brown SL Mikels JA Conway AM. Happiness unpacked: positive emotions increase life satisfaction by building resilience. Emotion. (2009) 9:361–8. 10.1037/a0015952 19485613 PMC3126102

[19] Fredrickson BL. The broaden-and-build theory of positive emotions. Philos Trans R Soc Lond B Biol Sci. (2004) 359:1367–78. 10.1098/rstb.2004.1512 15347528 PMC1693418

[20] Fredrickson BL Cohn MA Coffey KA Pek J Finkel SM. Open hearts build lives: positive emotions, induced through loving-kindness meditation, build consequential personal resources. J Pers Soc Psychol. (2008) 95:1045–62. 10.1037/a0013262 18954193 PMC3156028

[21] Forsman AK Wahlbeck K Aaro LE Alonso J Barry MM Brunn M. Research priorities for public mental health in Europe: recommendations of the ROAMER project. Eur Jo Public Health. (2015) 25:249–54. 10.1093/eurpub/cku232 25678606

[22] Räsänen P Lappalainen P Muotka J Tolvanen A Lappalainen R. An online guided ACT intervention for enhancing the psychological wellbeing of University students: a randomized controlled clinical trial. Behav Res Ther. (2016) 78:30–42. 10.1016/j.brat.2016.01.001 26848517

[23] Schotanus-Dijkstra M Drossaert CHC Pieterse ME Boon B Walburg JA Bohlmeijer ET. An early intervention to promote well-being and flourishing and reduce anxiety and depression: a randomized controlled trial. Internet Interv. (2017) 9:15–24. 10.1016/j.invent.2017.04.002 30135833 PMC6096189

[24] Haga SM Kinser P Wentzel-Larsen T Lisøy C Garthus-Niegel S Slinning K. Mamma Mia – a randomized controlled trial of an internet intervention to enhance subjective well-being in perinatal women. J Positive Psychol. (2020):1–9. 10.1080/17439760.2020.1738535

[25] Monteiro F Pereira M Canavarro MC Fonseca A. Be a Mom's efficacy in enhancing positive mental health among postpartum women presenting low risk for postpartum depression: results from a pilot randomized trial. Int J Environ Res Public Health. (2020) 17:4679. 10.3390/ijerph17134679 32610640 PMC7370106

[26] Peter T Roberts LW Dengate J. Flourishing in life: an empirical test of the dual continua model of mental health and mental illness among Canadian University students. Int J Ment Health Promotion. (2011) 13:13–22. 10.1080/14623730.2011.9715646

[27] Dreger S Buck C Bolte G. Material, psychosocial and sociodemographic determinants are associated with positive mental health in Europe: a cross-sectional study. BMJ Open. (2014) 4:e005095. 10.1136/bmjopen-2014-005095 24871540 PMC4039806

[28] Hu T Zhang D Wang J Mistry R Ran G Wang X. Relation between emotion regulation and mental health: a meta-analysis review. Psychol Rep. (2014) 114:341–62. 10.2466/03.20.PR0.114k22w4 24897894

[29] Kraiss JT Ten Klooster PM Moskowitz JT Bohlmeijer ET. The relationship between emotion regulation and well-being in patients with mental disorders: a meta-analysis. Compr Psychiatry. (2020) 102:152189. 10.1016/j.comppsych.2020.152189 32629064

[30] Zessin U Dickhauser O Garbade S. The relationship between self-compassion and well-being: a meta-analysis. Appl Psychol. (2015) 7:340–64. 10.1111/aphw.12051 26311196

[31] Fledderus M Bohlmeijer ET Pieterse ME. Does experiential avoidance mediate the effects of maladaptive coping styles on psychopathology and mental health? Behav Modif. (2010) 34:503–19. 10.1177/0145445510378379 20660354

[32] Neff KD. Self-compassion: an alternative conceptualization of a healthy attitude toward oneself. Self Identity. (2003) 2:85–101. 10.1080/15298860309032

[33] Trompetter HR de Kleine E Bohlmeijer ET. Why does positive mental health buffer against psychopathology? An exploratory study on self-compassion as a resilience mechanism and adaptive emotion regulation strategy. Cogn Ther Res. (2017) 41:459–68. 10.1007/s10608-016-9774-0 28515539 PMC5410199

[34] Schotanus-Dijkstra M Pieterse ME Drossaert CHC Walburg JA Bohlmeijer ET. Possible mechanisms in a multicomponent email guided positive psychology intervention to improve mental well-being, anxiety and depression: a multiple mediation model. J Positive Psychol. (2019) 14:141–55. 10.1080/17439760.2017.1388430

[35] Sirois FM Bögels S Emerson L-M. Self-compassion improves parental well-being in response to challenging parenting events. J Psychol. (2019) 153:327–41. 10.1080/00223980.2018.1523123 30376651

[36] Hayes SC Luoma JB Bond FW Masuda A Lillis J. Acceptance and commitment therapy: model, processes and outcomes. Behav Res Ther. (2006) 44:1–25. 10.1016/j.brat.2005.06.006 16300724

[37] Fledderus M Bohlmeijer ET Smit F Westerhof GJ. Mental health promotion as a new goal in public mental health care: a randomized controlled trial of an intervention enhancing psychological flexibility. Am J Public Health. (2010) 100:2372. 10.2105/AJPH.2010.196196 20966360 PMC2978181

[38] Ostergaard T Lundgren T Zettle RD Landro NI Haaland VO. Psychological flexibility in depression relapse prevention: processes of change and positive mental health in group-based ACT for residual symptoms. Front Psychol. (2020) 11:528. 10.3389/fpsyg.2020.00528 32292369 PMC7119364

[39] Gratz KL Roemer L. Multidimensional assessment of emotion regulation and dysregulation: development, factor structure, and initial validation of the difficulties in emotion regulation scale. J Psychopathol Behav Assess. (2004) 26:41–54. 10.1023/B:JOBA.0000007455.08539.94

[40] Gutierrez-Zotes A Labad J Martin-Santos R Garcia-Esteve L Gelabert E Jover M. Coping strategies for postpartum depression: a multi-centric study of 1626 women. Arch Women's Ment Health. (2016) 19:455–61. 10.1007/s00737-015-0581-5 26399872

[41] Haga SM Ulleberg P Slinning K Kraft P Steen TB Staff A. A longitudinal study of postpartum depressive symptoms: multilevel growth curve analyses of emotion regulation strategies, breastfeeding self-efficacy, and social support. Arch Women's Ment Health. (2012) 15:175–84. 10.1007/s00737-012-0274-2 22451329

[42] Fonseca A Gorayeb R Canavarro MC. Womens help-seeking behaviours for depressive symptoms during the perinatal period: socio-demographic and clinical correlates and perceived barriers to seeking professional help. Midwifery. (2015) 31:1177–85. 10.1016/j.midw.2015.09.002 26433622

[43] Dennis CL Chung-Lee L. Postpartum depression help-seeking barriers and maternal treatment preferences: a qualitative systematic review. Birth. (2006) 33:323–31. 10.1111/j.1523-536X.2006.00130.x 17150072

[44] Bayrampour H Trieu J Tharmaratnam T. Effectiveness of eHealth interventions to reduce perinatal anxiety: a systematic review and meta-analysis. J Clin Psychiatry. (2019) 80:18r12386. 10.4088/JCP.18r12386 30688418

[45] Ashford MT Olander EK Ayers S. Computer- or web-based interventions for perinatal mental health: a systematic review. J Affect Disord. (2016) 197:134–46. 10.1016/j.jad.2016.02.057 26991368

[46] Huppert FA. A new approach to reducing disorder and improving well-being. Pers Psychol Sci. (2009) 4:108–11. 10.1111/j.1745-6924.2009.01100.x 26158844

[47] Fonseca A Pereira M Araújo-Pedrosa A Gorayeb R Ramos MM Canavarro MC. Be a mom: formative evaluation of a web-based psychological intervention to prevent postpartum depression. Cogn Behav Pract. (2018) 25:473–95. 10.1016/j.cbpra.2018.02.002

[48] Fonseca A Monteiro F Alves S Gorayeb R Canavarro MC. Be a mom, a web-based intervention to prevent postpartum depression: the enhancement of self-regulatory skills and its association with postpartum depressive symptoms. Front Psychol. (2019) 10:265. 10.3389/fpsyg.2019.00265 30873060 PMC6401984

[49] Fonseca A Alves S Monteiro F Gorayeb R Canavarro MC. Be a mom, a web-based intervention to prevent postpartum depression: results from a pilot randomized controlled trial. Behav Ther. (2019). 10.1016/j.beth.2019.09.007 32586434

[50] Alves S Fonseca A Canavarro MC Pereira M. Preliminary psychometric testing of the Postpartum Depression Predictors Inventory-Revised (PDPI-R) in Portuguese women. Matern Child Health J. (2018) 22:571–8. 10.1007/s10995-017-2426-5 29327321

[51] Alves S Fonseca A Canavarro MC Pereira M. Predictive validity of the Postpartum Depression Predictors Inventory-Revised (PDPI-R): A longitudinal study with Portuguese women. Midwifery. (2019) 69:113–20. 10.1016/j.midw.2018.11.006 30496938

[52] Monteiro F Fonseca A Pereira M Canavarro MC. Measuring positive mental health in the postpartum period: the bifactor structure of the Mental Health Continuum-Short Form in Portuguese women. Assessment. (2020) 28:1434–44. 10.1177/1073191120910247 32167379

[53] Castilho P Pinto-Gouveia J Duarte J. Evaluating the multifactor structure of the long and short versions of the self-compassion scale in a clinical sample. J Clin Psychol. (2015) 71:856–70. 10.1002/jclp.22187 25907562

[54] Pinto-Gouveia J Gregório S Dinis A Xavier A. Experiential avoidance in clinical and non-clinical samples: AAQ-II Portuguese version. Int J Psychol Psychol Ther. (2012) 12: 139–56.

[55] Moreira H Gouveia MJ Canavarro MC. A bifactor analysis of the Difficulties in Emotion Regulation Scale - Short Form (DERS-SF) in a sample of adolescents and adults. Curr Psychol. (2020). 10.1007/s12144-019-00602-5

[56] Schulz KF Altman DG Moher D Group C. CONSORT 2010 statement: updated guidelines for reporting parallel group randomised trials. BMJ. (2010) 340:c332. 10.1136/bmj.c332 20332509 PMC2844940

[57] Siddiqui O Hung HM O'Neill R. MMRM vs. LOCF: a comprehensive comparison based on simulation study and 25 NDA datasets. J Biopharmaceutical Stat. (2009) 19:227–46. 10.1080/10543400802609797 19212876

[58] Hayes AF Rockwood NJ. Conditional process analysis: concepts, computation, and advances in the modeling of the contingencies of mechanisms. Am Behav Sci. (2020) 64:19–54. 10.1177/0002764219859633

[59] Bauer DJ Preacher KJ Gil KM. Conceptualizing and testing random indirect effects and moderated mediation in multilevel models: new procedures and recommendations. Psychol Methods. (2006) 11:142–63. 10.1037/1082-989X.11.2.142 16784335

[60] Hofmann SG Curtiss JE Hayes SC. Beyond linear mediation: toward a dynamic network approach to study treatment processes. Clin Psychol Rev. (2020) 76:101824. 10.1016/j.cpr.2020.101824 32035297 PMC7137783

[61] Beck CT. Revision of the postpartum depression predictors inventory. J Obstet Gynecol Neonatal Nurs. (2002) 31:394–402. 10.1111/j.1552-6909.2002.tb00061.x 12146928

[62] Sutherland J. Mothering, guilt and shame. Sociol Compass. (2010) 4:310–21. 10.1111/j.1751-9020.2010.00283.x

[63] Dennis CL Dowswell T. Psychosocial and psychological interventions for preventing postpartum depression. Cochrane Database Syst Rev. (2013). CD001134. 10.1002/14651858.CD001134.pub3 PMC11936315 23450532

[64] Benoy C Knitter B Schumann I Bader K Walter M Gloster AT. Treatment sensitivity: its importance in the measurement of psychological flexibility. J Contextual Behav Sci. (2019) 13:121–5. 10.1016/j.jcbs.2019.07.005

[65] Levin ME Haeger JA Pierce BG Twohig MP. Web-based acceptance and commitment therapy for mental health problems in college students: a randomized controlled trial. Behav Modif. (2017) 41:141–62. 10.1177/0145445516659645 27440189

[66] Kato T. Development of the coping flexibility scale: evidence for the coping flexibility hypothesis. J Couns Psychol. (2012) 59:262–73. 10.1037/a0027770 22506909

[67] Felder JN Lemon E Shea K Kripke K Dimidjian S. Role of self-compassion in psychological well-being among perinatal women. Arch Womens Ment Health. (2016) 19:687–90. 10.1007/s00737-016-0628-2 27138783

[68] Räsänen P Muotka J Lappalainen R. Examining mediators of change in wellbeing, stress, and depression in a blended, Internet-based, ACT intervention for University students. Internet Interv. (2020) 22:100343. 10.1016/j.invent.2020.100343 32995301 PMC7508697

[69] Wersebe H Lieb R Meyer AH Hofer P Gloster AT. The link between stress, well-being, and psychological flexibility during an acceptance and commitment therapy self-help intervention. Int J Clin Health Psychol. (2018) 18:60–8. 10.1016/j.ijchp.2017.09.002 30487911 PMC6220909

[70] Lee EW Denison FC Hor K Reynolds RM. Web-based interventions for prevention and treatment of perinatal mood disorders: a systematic review. BMC Pregnancy Childbirth. (2016) 16:38. 10.1186/s12884-016-0831-1 26928898 PMC4770541

[71] Winer ES Cervone D Bryant J McKinney C Liu RT Nadorff MR. Distinguishing mediational models and analyses in clinical psychology: atemporal associations do not imply causation. J Clin Psychol. (2016) 72:947–55. 10.1002/jclp.22298 27038095

